# Evolutionary adaptation of an RNA bacteriophage to the simultaneous increase in the within-host and extracellular temperatures

**DOI:** 10.1038/s41598-018-26443-z

**Published:** 2018-05-24

**Authors:** Ester Lázaro, María Arribas, Laura Cabanillas, Ismael Román, Esther Acosta

**Affiliations:** 1 Department of Molecular Evolution, Centro de Astrobiología (CSIC-INTA), Ctra de Ajalvir, Km 4, 28850 Torrejón de Ardoz, Madrid, Spain; 2 Grupo Interdisciplinar de Sistemas Complejos (GISC), Madrid, Spain

## Abstract

Bacteriophages are the most numerous biological entities on Earth. They are on the basis of most ecosystems, regulating the diversity and abundance of bacterial populations and contributing to the nutrient and energy cycles. Bacteriophages have two well differentiated phases in their life cycle, one extracellular, in which they behave as inert particles, and other one inside their hosts, where they replicate to give rise to a progeny. In both phases they are exposed to environmental conditions that often act as selective pressures that limit both their survival in the environment and their ability to replicate, two fitness traits that frequently cannot be optimised simultaneously. In this study we have analysed the evolutionary ability of an RNA bacteriophage, the bacteriophage Qβ, when it is confronted with a temperature increase that affects both the extracellular and the intracellular media. Our results show that Qβ can optimise its survivability when exposed to short-term high temperature extracellular heat shocks, as well as its replicative ability at higher-than-optimal temperature. Mutations responsible for simultaneous adaptation were the same as those selected when adaptation to each condition proceeded separately, showing the absence of important trade-offs between survival and reproduction in this virus.

## Introduction

Viruses play a relevant role in ecosystems. They are the most abundant biological entities across all habitats, from the oceans to the human body, and there is probably no species that cannot be infected by some kind of virus. Within viruses, bacteriophages –or just phages for simplicity- are the most numerous and most widely distributed, outnumbering their hosts by a factor between 10 and 100. In ecosystems dominated by microorganisms they act as one of the main causes of mortality, shaping biological diversity and contributing to the nutrient and energy cycles, which has a direct consequence on how food webs structure and on the recycling of biogeochemical elements on Earth^1–4^. Thus, it is easy to imagine that any factor that alters the size and structure of phage populations may have a great impact on the composition and structure of the entire ecosystem^5^.

Temperature is one of the environmental conditions with a greater influence on phages. It represents a selective pressure that acts on the two phases of their life cycle, the period between infections or extracellular phase, in which phages behave as inert particles exposed to the physical-chemical conditions of the environment^6–8^, and the intracellular period, when they replicate to give rise to a progeny. Life-history theory suggests that traits for reproduction and survival cannot be simultaneously optimised^9^, meaning that improvements in virus within-host reproduction are frequently associated to reductions in the stability in the external medium and vice versa. As a result of this kind of trade-off, adaptation to increased temperature, could be greatly hampered in phages. A general property of viruses, and of RNA viruses in particular, is that genomic replication takes place with very high mutation rates^10,11^, which leads to the generation of highly heterogeneous populations, containing a wide diversity of genotypes, some of which may have increased performance in new environments. This fact accelerates evolution enormously^12,13^ and can make it possible to find adaptive solutions to selective pressures that act on different traits that are difficult to optimise simultaneously. The generation and permanence of the beneficial genotypes also depends on the population size^14,15^, which can be greatly reduced under strong selective pressures. Thus, big and sudden temperature changes would be less compatible with adaptation than mild or gradual ones, entailing that the pattern of temperature change will also determine the adaptive capacity of populations to this condition^16–18^. Currently, the study of adaptation to high temperature represents a topic of special relevance, due to the process of global warming that our planet is experiencing^19–21^. Some species are responding to this challenge by shifting their geographical distribution or changing the time of reproduction. However, phages are entirely dependent on factors beyond their control for their dispersal and multiplication, causing that their adaptation is mainly based on evolutionary rescue^22–24^. This fact adds interest to the study of the conditions under which phage adaptation to increased temperature can take place.

In this work we have carried out experimental evolution of an RNA phage that infects *E. coli*, the bacteriophage Qβ, to study its capacity to simultaneously adapt to increased extracellular and intracellular temperatures. This phage has a small genome (4217 nucleotides) that makes it easy to establish phenotype-genotype relationships. It encodes only 4 proteins: the A2 protein for bacterial lysis and entry; the coat protein; the A1 protein, present in low amount in the capsid and that is expressed through incorrect reading of the stop codon of the coat protein; and the replicase^25^. Bacteriophage Qβ replicates with very high mutation rate^26^ and forms highly diverse populations in which adaptation proceeds rapidly. These facts, together with the ease of its propagation, make it a suitable system to perform evolution studies in a lab-controlled environment. It was previously demonstrated that Qβ can adapt to increases in the replication temperature^27–29^, and also to short-term 52 °C heat shocks in the extracellular medium^30^. However, it is unknown whether the phage can adapt to both circumstances simultaneously, something that may be frequent in nature. Whereas the within-host temperature cannot exceed the maximum value that is compatible with host viability, the extracellular temperature can reach higher values, which can reduce the population size, becoming a factor that limits subsequent adaptation. In order to investigate the limits of adaptation to high temperature in Qβ we have chosen two extreme values: 60 °C for the extracellular heat shocks and 43 °C for replication. The specific objectives of our study are: i) to study whether bacteriophage Qβ can adapt to more extreme extracellular temperatures than those previously reported, ii) to determine the contribution of generated versus extant genetic diversity on this adaptation, iii) to investigate whether increased survival at high extracellular temperature presents a trade-off with replication, iv) to identify the patterns of change that are compatible with simultaneous adaptation to the increase in both the extracellular and intracellular temperatures, and v) to study whether simultaneous adaptation occurs through the same genetic changes as adaptation to each condition separately.

## Methods

### Virus populations, bacteria and standard procedures for infection

Bacteriophage Qβ was propagated in *Escherichia coli*, strain Hfr (Hayes) in NB medium (8 g/l Nutrient Broth from Merck and 5 g/l NaCl). Infections in liquid medium were carried out using fresh log-phase *E. coli* cultures with an OD_550_ between 0.6 and 0.8, which were infected at the multiplicity of infection (moi) indicated in each experiment. After 2 h of incubation either at the optimal (37 °C) or sub-optimal temperatures with good aeration (250 rpm), cultures were treated with 1/20 volume of chloroform for 15 min at 37 °C with shaking (300 rpm). Virus supernatants were harvested upon centrifugation at 13000 × g. Virus titres were determined by plaque assay and expressed as the number of plaque forming units (pfu) per ml of the phage suspension.

The plasmid pBRT7Qβ, which contains the cDNA of bacteriophage Qβ cloned in the plasmid pBR322^31,32^ was used to transform *E. coli* DH5-α. The supernatant of an overnight culture obtained from a transformed colony was used to isolate a biological clone [virus Qβ(wt)] that corresponded to the virus progeny contained in a lytic plaque. The virus Qβ(wt) showed no mutations relative to the Qβ cDNA sequence cloned in the plasmid pBRT7Qβ. This virus was used to infect an *E. coli* culture as described above, using a multiplicity of infection (moi) of 0.01 pfu/cell in a volume of 10 ml (containing ~10^9^ bacteria). After 2 h of incubation at 37 °C, the virus supernatant was collected, and approximately 10^7^ pfu of the phage suspension were used to infect a fresh *E. coli* culture, keeping the moi around 0.01 pfu/cell. The process was repeated for 20 serial transfers to obtain a heterogeneous virus population [Qβ(wt-p20)], which presented two polymorphisms [G1773(G + A) and A2187(C + A)] relative to the sequence of the virus Qβ(wt).

The plasmid pBRT7Qβ was also used to engineer a single-mutant virus [Qβ(A1956G)] containing the substitution A1956G, which was selected in the experimental evolution assays carried out in this work. The single mutant was used to test the effect of substitution A1956G in the context of the wild-type virus. Mutagenesis was carried out using the QuickChange II Site-Directed Mutagenesis Kit (Stratagene) and the primers 5′CTTAGACTCGTCTGAGGTGACTGTTTACGGAGACGA-3′ and its complementary. The procedures to build and isolate the site-directed mutant have been previously described^33,34^. A lytic plaque produced by this mutant was picked and the presence of the desired mutation verified.

### Evolutionary lineages of bacteriophage Qβ

**Figure 1:**
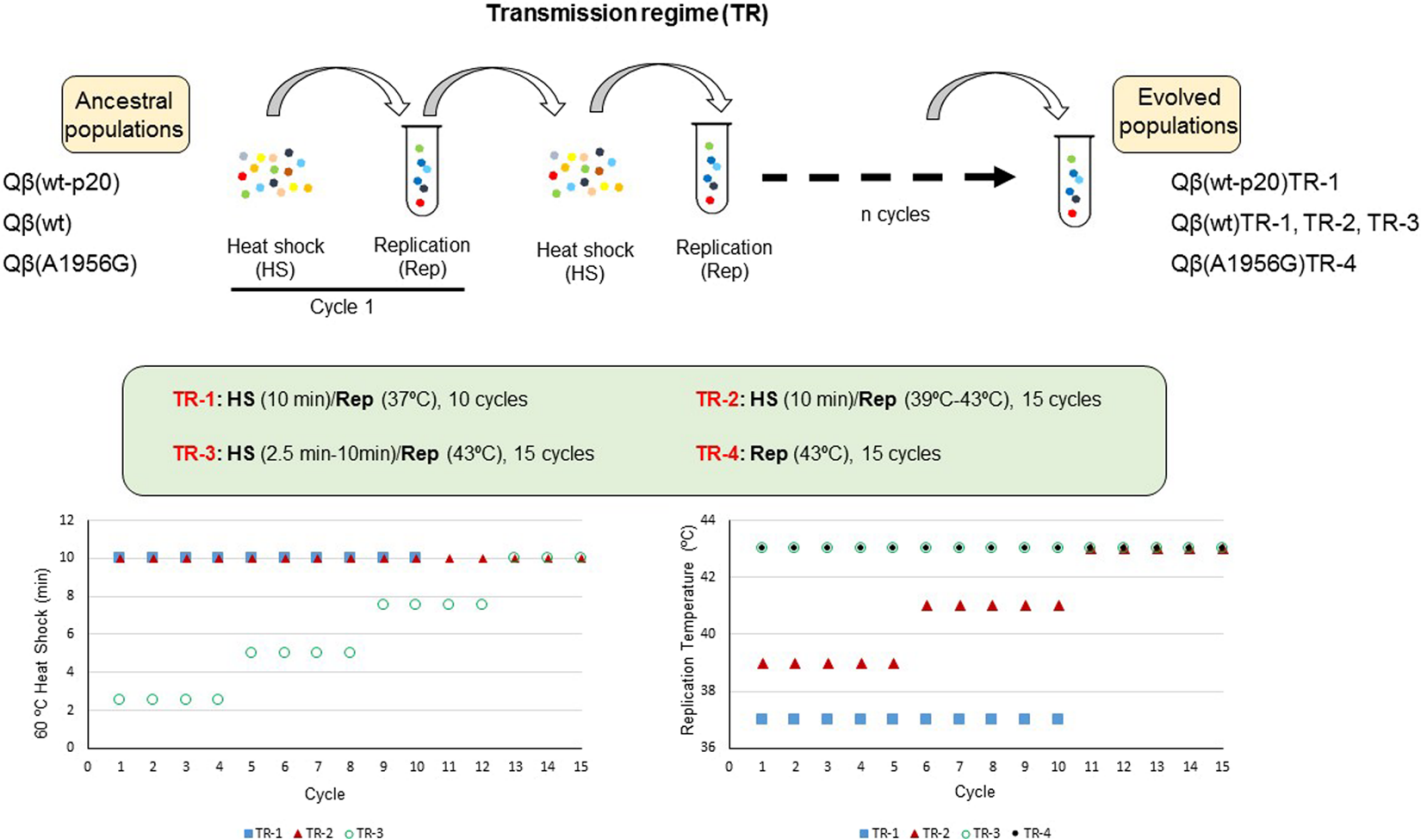
Evolutionary lineages of bacteriophage Qβ. Evolved populations Qβ(wt-p20)TR-1, Qβ(wt)TR-1, TR-2, and TR-3; and Qβ(A1956G)TR-4 were obtained upon propagation of the corresponding ancestral populations [Qβ(wt-p20), Qβ(wt), or Qβ(A1956G)] through the indicated transmission regimes (TR). TR-1 consisted of 10 cycles in which 10 min 60 °C heat shocks alternated with replication at 37 °C. TR-2 corresponded to 15 cycles of 10 min 60 °C heat shocks alternated with replication at an increased temperature that ranged from 39 °C to 43 °C. TR-3 consisted of 15 cycles in which 60 °C heat shocks (from 2.5 min to 10 min) alternated with replication at 43 °C. TR-4 was used only for population Qβ(A1956G), and consisted in its propagation through 15 transfers at 43 °C in the absence of heat shocks. All lineages were duplicated. Graphics at the bottom of the figure represent the pattern of change either in the duration of the heat shock or the replication temperature for each particular transmission regime.

The viruses Qβ(wt-p20), Qβ(wt), and Qβ(A1956G) were the founders of all the evolutionary lineages used in this work. Evolved populations Qβ(wt-p20)TR-1, Qβ(wt)TR-1, TR-2, and TR-3; and Qβ(A1956G)TR-4 were obtained upon propagation of the corresponding ancestral populations through different transmission regimes (Fig. 1). Transmission regimes 1, 2, 3 (TR-1, TR-2, and TR-3) consisted of repeated cycles of 60 °C extracellular heat shock alternated with replication at either optimal or non-optimal temperature (Fig. 1). In all cases the first cycle was initiated with 10^7^–10^8^ pfus that were heated at 60 °C in a thermoblock for the time indicated, in a final volume of 1 ml of Phage Buffer (PB) (1 g/l gelatine, 0.05 M Tris–HCl, pH 7.5, and 0.01 M MgCl_2_). After each heat shock, the sample was immediately chilled on ice, and 100 µl, containing a maximum of 10^7^ pfu (otherwise the culture was diluted), were used to infect a fresh *E. coli* culture containing ~10^8^ bacteria in a final volume of 1 ml of NB. The culture was incubated for 2 h with shaking (250 rpm) at the temperature indicated in each case, and processed with chloroform to obtain a virus supernatant which was diluted 1:10 in PB before being subjected to the next heat shock, again in a final volume of 1 ml. Virus titres were determined at each cycle both after the heat shock and after the replication to ensure that the virus was not extinguished and that the moi was always below 1. In the case of TR-1, heat shocks had the maximum duration (10 min at 60 °C) and virus replication took place at optimal temperature (37 °C). TR-2 involved a gradual increase in the replication temperature (39 °C for cycles 1 to 5, 41 °C for cycles 6 to 10, and 43 °C for cycles 11 to 15) whereas the duration of the heat shock was kept constant (10 min). In the case of TR-3, the replication temperature was kept constant at 43 °C, whereas the duration of the 60 °C heat shock was increased gradually (2.5 min for cycles 1 to 4; 5 min for cycles 5 to 8; 7.5 min for cycles 9 to 12; and 10 min for cycles 13 to 15. Transmission regime 4 (TR-4) involved virus replication through 15 serial transfers which were carried out infecting 1 ml *E. coli* cultures, containing 10^8^ cells at an moi of 0.1. Cultures were incubated at 43 °C for 2 h, after which the virus supernatant was collected as described above and used to initiate the next transfer in the absence of intermediate heat shocks.

Population Qβ(wt-p20) was also used to obtain a control lineage, which was propagated for 10 cycles consisting of 10 min heat shocks at 37 °C alternated with replication (2 h, 37 °C). Transmission protocol was similar to TR-1, with the only difference of the heat shock temperature.

### Fitness determinations

Triplicate liquid cultures containing 10^8^ bacteria growing in exponential phase were inoculated with 10^4^ pfu in a final volume of 1 ml. After two hours of incubation at the temperature indicated, the virus supernatants were collected as described above, and titrated to estimate the virus yield, which was used as a surrogate of fitness. Virus fitness was expressed as the number of doublings per two hours, and was calculated as log_2_[(*N*_*f*_ - *N*_0_)/*N*_0_], where *N*_0_ was the initial input of virus and *N*_*f*_ was the number of progeny pfu. Previous experiments carried out in our group showed that, when infections were carried out under these conditions, the bacteria concentration was kept in large excess over that of phages, during the two hours of the assay.

### RNA extraction, cDNA synthesis, PCR amplification, and nucleotide sequencing

Viral RNA was prepared following standard procedures to determine the consensus sequence (from nucleotide 250 to nucleotide 4180). RNAs were used for cDNA synthesis with the avian myeloblastosis virus reverse transcriptase (Promega), followed by PCR amplification using Expand high-fidelity DNA polymerase (Roche). PCR products were column purified (Qiagen) and subjected to standard Sanger sequencing. The oligonucleotide primers used for sequencing have been previously described^33,34^. Sequences were aligned with Clustal W. Mutations relative to the sequence of the cDNA of bacteriophage Qβ cloned in the plasmid pBR322^31,32^ were identified using BioEdit.

### Data availability

The datasets generated and/or analysed during the current study are available from the corresponding author on reasonable request. Nucleotide sequences have been deposited in NCBI GenBank (accession numbers MH108093-MH108104).

## Results

### Adaptation of bacteriophage Qβ to high temperatures in the extracellular environment

**Figure 2:**
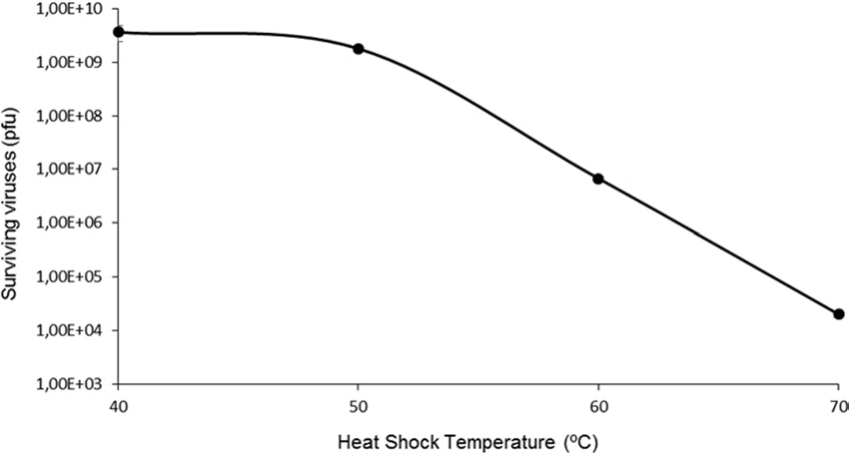
Sensitivity of bacteriophage Qβ to extracellular heat shocks. Population Qβ(wt-p20) (5 × 10^9^ pfu in 1 ml of phage buffer) was subjected to a single 10 min heat shock at the temperatures indicated in the x-axis, and the number of surviving virus was estimated through plaque assay. Each point represents the average of two independent determinations. Error bars showing the standard deviations are included in the figure, although they are too small to be visible.

Previously to the adaptation experiments, we determined the sensitivity of bacteriophage Qβ to short-term exposure to high temperature in the extracellular environment. To do that, we subjected the population Qβ(wt-p20) (see Methods) to 10 min heat shocks at a temperature which ranged from 40 °C to 70 °C, and estimated the number of surviving viruses through plaque assay. The results obtained showed that exposure to 40 °C and 50 °C had low impact on virus viability, whereas similar exposure to 60 °C or 70 °C decreased virus infectivity about 3 and 5 orders of magnitude respectively (Fig. 2).

**Figure 3:**
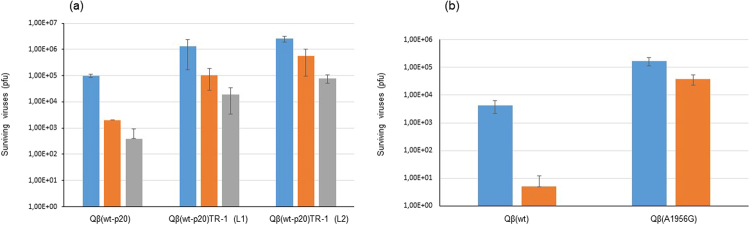
Sensitivity of different Qβ populations to extracellular 60 °C heat shocks. (**a**) Number of surviving viruses recovered after exposing to 60 °C 10^7^ pfus of the initial population Qβ(wt-p20) and of the two replicas (L1 and L2) obtained upon its propagation through transmission regime 1 (Fig. 1). (**b**) Number of surviving viruses recovered after exposing to 60 °C 5 × 10^5^ pfu of the virus Qβ(wt) and of the single mutant Qβ(A1956G). The duration of the heat shock was 10 min (blue bars), 20 min (orange bars), and 30 min (grey bars). The bars represent the average of two independent determinations. The error bars correspond to the standard deviation.

To find out whether bacteriophage Qβ was able to increase its resistance to harmful extracellular temperatures through evolutionary adaptation, we propagated population Qβ(wt-p20) through a protocol that consisted in the alternation of 10 min heat shocks at 60 °C with replication in *E. coli* under optimal conditions (2 h, 37 °C) (TR-1 in Fig. 1). Replicate populations obtained after 10 cycles [Qβ(wt-p20)TR-1, lineages 1 and 2] increased their resistance to the exposure to 60 °C in the extracellular environment (Fig. 3a), showing that Qβ can rapidly adapt to this condition. The analysis of the consensus sequences of the adapted populations showed the presence of two mutations [A2187C (S281R in A1), and G1773(G + A) (G143R in A1)] which were already present in the ancestral population Qβ(wt-p20) (see Methods) although with lower representation of the mutated nucleotide. In addition, both replicate lineages showed the fixation of a new non-synonymous mutation A1956G (K204E in the A1 protein), which was the only change with respect to the consensus sequence of the ancestral population. A control experiment in which population Qβ(wt-p20) was transferred through a protocol similar to TR-1, except that the heat shocks took place at 37 °C, showed that under this condition the only detectable mutations were G1773(G + A) and A2187C, strongly suggesting that A1956G was selected in response to the 60 °C heat shock. To further assess whether this substitution was responsible for the increased resistance of the evolved populations to the extracellular heat shock, we introduced it in the Qβ infectious clone through site-directed mutagenesis (see Methods). The single mutant obtained, Qβ(A1956G) displayed higher resistance to 60 °C heat shocks than the virus Qβ(wt), obtained upon expression of the infectious clone non-subjected to mutagenesis (Fig. 3b), clearly indicating that substitution A1956G confers increased resistance to high extracellular temperature in Qβ.

A possible explanation for the rapid adaptation of population Qβ(wt-p20) to 60 °C heat shocks is that, since this population was heterogeneous, it already contained some mutants conferring increased resistance to high extracellular temperatures. These variants would increase their frequency upon propagation through repeated cycles of heat shock and replication. In this way adaptation would have taken place without the *de novo* generation of resistance mutations. To analyse the influence of the pre-existent genetic diversity on the adaptation of Qβ to high extracellular temperature, we propagated the clonal virus Qβ(wt) (see Methods) through the same protocol used for population Qβ(wt-p20) (TR-1 in Fig. 1). The two replicate lineages obtained [Qβ(wt)TR-1, L1 and L2] showed the fixation in their consensus sequences of the same substitution A1956G that had been selected when evolution was initiated with the heterogeneous population Qβ(wt-p20), with no additional nucleotide changes. Since the virus Qβ(wt) was isolated as a lytic plaque (see Methods), and lytic plaques are usually generated upon replication of single genomes for a short number of replication rounds, it is expected that it contains a mutant spectrum of very low genetic diversity, in which mutants with selective advantages at high extracellular temperature are probably not present. Thus, substitution A1956G was most likely generated *de novo* in the course of the evolution experiment.

### Replicative ability of the mutant Qβ(A1956G) at optimal and suboptimal temperatures

To analyse whether the presence of substitution A1956G has some influence on the replicative ability of Qβ, we determined the virus yields produced upon replication of the single mutant Qβ(A1956G) and the virus Qβ(wt) at three different temperatures, the optimal (37 °C) and two suboptimal (30 °C and 43 °C). Our results showed that the mutant has similar fitness values to the wild-type virus at 37 °C and 30 °C (*P* = 0.17 and *P* = 0.23 respectively; Student’ *t* test for the difference of means), and significantly higher fitness than the wild type at 43 °C (*P* = 0.02, Student’ *t* test for the difference of means) (Table 1). Thus, the increased resistance to high temperature in the extracellular environment provided by substitution A1956G does not entail a trade-off with replication at 37 °C and 30 °C. Furthermore, the presence of this substitution seems to be associated to a significant improvement in the replicative ability at higher-than-optimal temperature, although this improvement was too slight to recover the virus yields obtained at 30 °C and 37 °C.

**Table 1:** Fitness values of the clonal viruses Qβ(wt) and Qβ(A1956G) at different replication temperatures.

Temperature	Virus	
	Qβ(wt)	Qβ(A1956G)
37 °C	16.2 ± 0.1	16.5 ± 0.5 (*P* = 0.17)
30 °C	13.3 ± 0.3	13.5 ± 0.3 (*P* = 0.23)
43 °C	7.2 ± 0.3	8.8 ± 0.6 (*P* = 0.02)

The values represent the average and the standard deviation of three independent determinations carried out as described in Methods. The *P* value (Student’ *t* test) for the difference of means between the viruses Qβ(wt) and Qβ(A1956G) at each temperature is also indicated.

### Adaptation of bacteriophage Qβ to the increase in both extracellular and intracellular temperatures

The absence in Qβ of an apparent trade-off between the increase in the resistance to high extracellular temperature and the replicative ability at higher-than-optimal temperature (Table 1) suggests that this phage could be able to adapt simultaneously to both conditions. To analyse this issue, we subjected the virus Qβ(wt) to 10 min 60 °C heat shocks alternated with replication at 43 °C instead of 37 °C. We carried out several assays starting with different amount of viruses, and in all cases, after the second or the third heat shock the virus was extinguished. Adaptation under these conditions was further impaired due to an increase in virus sensitivity to the extracellular heating when virus supernatants obtained after replication in *E. coli* were directly exposed to 60 °C, an effect that disappeared when they were diluted at least ten times in phage buffer (data not shown). Unfortunately, the low titres obtained after replication at 43 °C forced us to expose the undiluted virus supernatants directly to the heat shock, which accelerated the virus decay.

**Figure 4:**
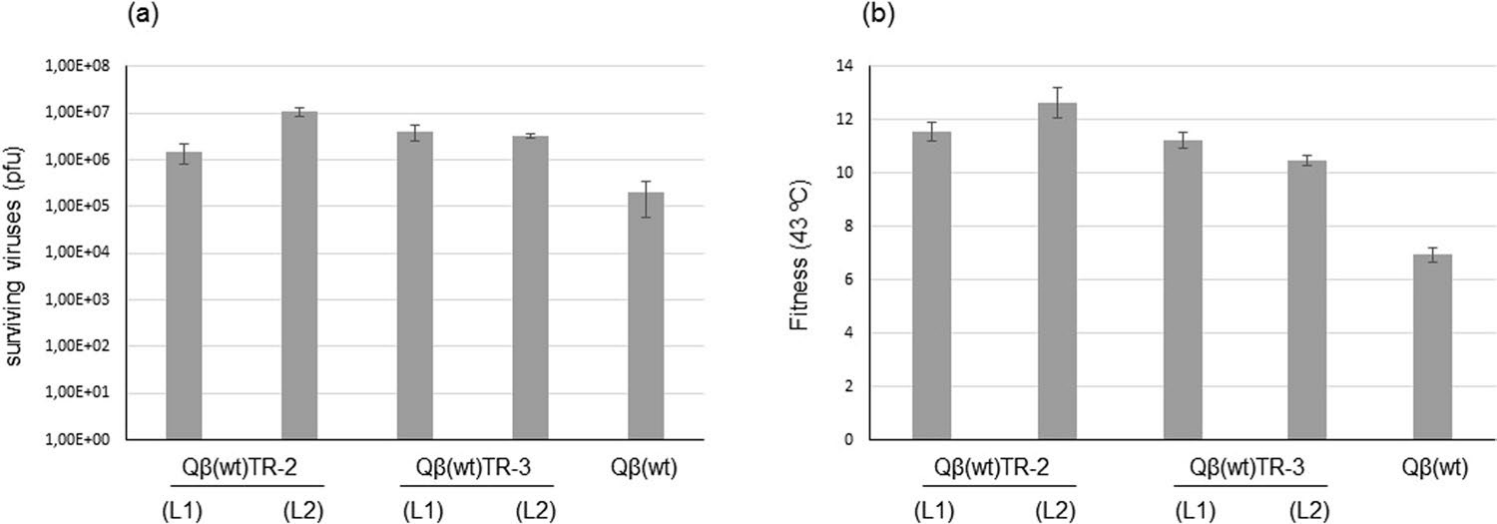
Characterization of Qβ populations evolved through a gradual increase in either the replication temperature or the duration of the extracellular heat shock. (**a**) Number of surviving viruses recovered after exposing to 60 °C for 10 min 10^7^ pfus of the ancestor population Qβ(wt) or the evolved populations obtained upon its propagation through transmission regimes 2 and 3 (Fig. 1). L1 and L2 represent replicate evolutionary lineages. The bars show the average of two independent determinations. The error bars correspond to the standard deviation. (**b**) Fitness at 43 °C of the same populations described in (**a**). The bars show the average of three independent determinations. The error bars correspond to the standard deviation. In all cases *P* values comparing fitness values of the wild type with each of the evolved populations were lower than 0.001 (Student’s t test for the difference of means).

To avoid reductions in the population size severe enough to preclude adaptation, we designed new transmission regimes in which one of the two selective pressures, either the replication temperature (TR-2 in Fig. 1) or the duration of the extracellular heat shock (TR-3 in Fig. 1), increased gradually until reaching the maximum value (43 °C for replication and 10 min for the heat shock). In each case the remaining selective pressure was always applied at the maximum value assayed. Each evolutionary lineage was propagated in replicate for a total of 15 cycles of heat shock followed by replication. The evolved populations were analysed to determine both the fitness value at 43 °C and the resistance to the heat shock (Fig. 4). Both patterns of change avoided extinction and allowed the virus to increase its performance under the two conditions.

Simultaneous adaptation to two different selective pressures could also be promoted if they were sequentially applied. To test this possibility, we propagated the single mutant Qβ(A1956G), which shows increased resistance to 60 °C heat shocks (Fig. 3b), through 15 serial transfers in which replication took place at 43 °C (TR-4 in Fig. 1). Evolved populations [Qβ(A1956G)TR-4_,_ lineages 1 and 2] kept substitution A1956G, and increased their fitness at 43 °C relative to the ancestor Qβ(A1956G) 1.5- and 1.4- folds respectively (*P* = 0.006 and *P* = 0.02 respectively; Student’ *t* test for the difference of means). Altogether, the results show that bacteriophage Qβ can adapt to both replication at 43 °C and exposure to 60 °C extracellular heat shocks, provided that adaptation to each temperature proceeds sequentially or at least one of the selective pressures increases gradually.

### Genetic changes in bacteriophage Qβ populations adapted to the increase in both extracellular and intracellular temperatures

The results of the current study allowed us to analyse whether joint adaptation to increased extracellular and intracellular temperatures took place through the same substitutions that were selected when Qβ was exposed to each selective pressure separately. We also analysed whether the adaptive pathways followed by populations depended on the pattern of change in the selective pressures. To investigate these questions, we determined the consensus sequences of the Qβ populations evolved through the different transmission regimes shown in Fig. 1, and compared the mutations acquired in each of them (Table 2). We found that A1956G was the only substitution that fixed in all evolutionary lineages. It was also the only substitution in common with those reported in a previous study in which Qβ was adapted to milder heat shocks (52 °C) alternating with replication at optimal temperature^30^. The result suggests that substitution A1956G is highly relevant for adaptation to 60 °C extracellular heat shocks, whether they are combined with replication at high temperature or not. Populations evolved through TR-2, TR-3, and TR-4 also acquired other mutations (fixed or polymorphic), most of them previously identified as responsible for adaptation to replicate at high temperature in Qβ (A1088G, U1295C, G1312A, C2201U, U2776C, and U3402C)^27,28^. In addition to those substitutions, some lineages presented others (U1766C, and A3798G) that have not been identified in previously published studies of adaptation to high temperatures or extracellular heat shocks.

**Table 2:** Nucleotide substitutions in the consensus sequences of the Qβ populations evolved at higher-than-optimal temperature.

Virus population	Nucleotide substitution								
	A1088G D342G A2	U1295C F411S A2	G1312A V417I A2	U1766C Syn A1	A1956G K204E A1	C2201U Syn A1	U2776C V141A Rep	U3402C S350P Rep	A3798G K482E Rep
Qβ(wt)TR-1 L1					G				
Qβ(wt)TR-1 L2					G				
Qβ(wt)TR-2 L1			A + G		G	U			
Qβ(wt)TR-2 L2			A + G		G	U			
Qβ(wt)TR-3 L1	G			C	G		U + C	U + C	A + G
Qβ(wt)TR-3 L2	A + G				G	U		C	
Qβ(A1956G) TR-4 L1	A + G		A + G		G			C	
Qβ(A1956G) TR-4 L2	A + G	U + C	A + G		G			C	

Virus populations are described in Fig. 1 and in Methods. L1 and L2 represent replicate evolutionary lineage. Nucleotide substitutions were determined with respect to the sequence of the Qβ cDNA cloned in pBR322^31,32^. Polymorphisms are indicated as the sum of the two nucleotides present at that position. Blank cells indicate the presence of the wild-type nucleotide. Below each nucleotide substitution we show the corresponding change of amino acid (Syn stands for synonymous substitutions) and the protein where it is located [A2, A1, or Replicase (Rep)].

## Discussion

This work illustrates the great adaptive ability of a phage when confronted with a harsh condition, a temperature increase, which targets the two phases of its life cycle: the inter-transmission period and the within-host period. Previous studies have shown the ability of phages to adapt to short-time heat shocks in the extracellular medium, provided that they are allowed to replicate at optimal temperature between each two consecutive heat shocks^30,35–37^. There are also several complementary studies focused on the adaptation of phages to replication at higher-than-optimal temperature in the absence of extracellular heat shocks^27–29,38–42^. However, in a real situation it is expected that the increase in the environmental temperature affects phages both when they are in the external medium and when they are replicating inside their hosts, which supposes a double challenge that may hamper adaptation, particularly if the improvement in one trait entails a cost in the other, as some studies suggest.

Increased resistance to high extracellular temperature usually takes place through the selection of mutations conferring higher capsid stability, which can have profound effects on capsid assembly kinetics through changes in the association energy between proteins, thereby reducing assembly efficiency and ultimately decreasing fitness. According to this, a study carried out with 16 phages infecting *E. coli* suggested the existence of a trade-off between survival in the environment and reproduction inside the host, similar to the trade-off between fertility and longevity described for higher organisms^43^. Another study carried out with vesicular stomatitis virus evolved through a regime that involved an increase in the time that the virus spent out of the host also selected for virus variants with increased extracellular survival and lower fecundity^44,45^. In contrast to these studies, our results show that increased resistance to extracellular heat shocks in Qβ did not entail reduced replicative ability at three different temperatures, including one higher than optimal. Our findings resemble those obtained with an ssDNA microvirid bacteriophage which showed a total absence of trade-offs after evolution through a protocol that alternated selection for increased growth rate at optimal temperature with selection for increased survival under low pH or increased temperature^46,47^.

The absence of trade-offs in our system may be a consequence of the simultaneous selection for increased growth rate and increased resistance to the exposure at 60 °C. Under this protocol, mutants with reduced performance under any of the two conditions might have been selected against, remaining undetectable. Substitution A1956G was the only common to all populations exposed to extracellular heat shocks. This finding, together with the increased resistance to heat shocks shown by the single mutant Qβ(A1956G) strongly supports its involvement in adaptation to this condition. The fact that this substitution was also the only one selected when inter-heat shock replication took place at optimal temperature, indicates that few solutions are possible for Qβ to optimise its resistance to high extracellular temperatures without paying a cost in its replicative capacity. The exclusivity of the selection of A1956G contrasts with the results of another study in which, following a protocol similar to ours but with a lower temperature heat shock (52 °C), a much larger number of mutations was detected^30^. It is possible that under the milder conditions used in this previous study, a higher repertoire of mutations showing no trade-offs between survival and reproduction were accessible. Increasing the temperature of the extracellular heat shock likely leads to the selection of mutations with a greater impact on capsid stability, possibly restricting the number of possible solutions that do not have a negative effect on fitness. However, we cannot exclude that other less frequent substitutions (e.g. involving a nucleotide transversion) could also increase the resistance to high temperature heat shocks. A larger number of experimental replicas would have been necessary to identify these substitutions. Substitution A1956G produces a very drastic change (Lysine 204 is substituted for Glutamic acid) in the A1 protein, which is produced when ribosomes occasionally read-through the leaky UGA termination codon of the coat protein gene and translation continues for another 600 nucleotides, resulting in a C-terminal extension of the coat protein^48^. The A1 protein is incorporated in 3–10 copies per virion and is essential for producing infectious virus particles, although its precise function is not known^49^. Substitution of the Lysine at position 204 for Glutamic acid supposes a change in the charge of the protein, which possibly has important consequences in its folding and in the interaction with other proteins. Determining the physicochemical properties of the Qβ particles assembled with the mutated A1 protein is beyond the scope of this study, although a better knowledge of this subject would be very useful to identify the precise function of the A1 protein and the molecular mechanisms that lead to the assembly of more stable capsids at high temperatures.

When 10 min 60 °C heat shocks were alternated with replication at 43 °C, Qβ populations were rapidly extinguished. These results illustrate a common limitation to adaptation that arises when the selective pressures are so strong that the population size is reduced to a level that prevents the generation and selection of adaptive mutations. Nevertheless, if any of the selective pressures was increased gradually, simultaneous adaptation to both of them became possible, showing the importance of the patterns of change in the environmental variables on the success of evolutionary rescue. Adaptation was also successful when a Qβ population with increased resistance to extracellular heat shocks (due to the presence of substitution A1956G) was propagated at 43 °C. This is a clear example in which sequential exposure to different selective pressures can facilitate adaptation. The fact that the effects of many mutations depend on the environment may cause that the pattern of change in the selective pressures influences the evolutionary trajectories. In this work, each lineage presents a higher degree of convergence with its replica than with the other lineages, suggesting an influence of the pattern of change in the selection of particular mutations.

Overall, this study supposes a thorough exploration of the patterns of temperature change and the evolutionary strategies that permit simultaneous adaptation to high extracellular and intracellular temperatures in a phage. Open questions for future work refer to the study of the role played by co-evolution between phages and their hosts in the success of evolutionary adaptation and the impact of mutations conferring increased thermostability on virus evolvability.
